# Investigation of Dengue Infection in Asymptomatic Individuals during a Recent Outbreak in La Réunion

**DOI:** 10.3390/v15030742

**Published:** 2023-03-14

**Authors:** Olga De Santis, Emilie Pothin, Nicolas Bouscaren, Seth R. Irish, Marie-Christine Jaffar-Bandjee, Luce Menudier, Julie Ramis, Cédric Schultz, Florence Lamaurt, Ania Wisniak, Antoine Bertolotti, Sarah Hafsia, Philippe Dussart, Laurence Baril, Patrick Mavingui, Antoine Flahault

**Affiliations:** 1 Inserm CIC1410, CHU de La Réunion, 97410 Saint Pierre, France; 2 Global Health Institute, University of Geneva, 1209 Geneva, Switzerland; 3 Swiss Tropical and Public Health Institute, 4002 Basel, Switzerland; 4 Department of Public Health, Faculty of Medicine, University of Basel, 4051 Basel, Switzerland; 5 Centre National de Référence Arbovirus Associé Réunion, CHU de La Réunion, 97400 Saint Denis, France; 6 Santé Publique France, 97400 Saint Denis, France; 7 UMR Processus Infectieux en Milieu Insulaire et Tropical, Cyroi, 97400 Saint Denis, France; 8 Institut de santé publique, d’épidémiologie et de développement (ISPED), Université de Bordeaux, 33000 Bordeaux, France; 9 Service des Maladies Infectieuses—Dermatologie, CHU de La Réunion, 97410 Saint Pierre, France; 10 Unité de Virologie, Institut Pasteur de Madagascar, Antananarivo 101, Madagascar; 11 Unité d’épidémiologie, Institut Pasteur de Madagascar, Antananarivo 101, Madagascar

**Keywords:** dengue, asymptomatic infections, La Réunion, cluster study, dengue outbreak

## Abstract

The number of dengue cases has increased dramatically over the past 20 years and is an important concern, particularly as the trends toward urbanization continue. While the majority of dengue cases are thought to be asymptomatic, it is unknown to what extent these contribute to transmission. A better understanding of their importance would help to guide control efforts. In 2019, a dengue outbreak in La Reunion resulted in more than 18,000 confirmed cases. Between October 2019 and August 2020, 19 clusters were investigated in the south, west, and east of the island, enabling the recruitment of 605 participants from 368 households within a 200 m radius of the home of the index cases (ICs). No active asymptomatic infections confirmed by RT-PCR were detected. Only 15% were possible asymptomatic dengue infections detected by the presence of anti-dengue IgM antibodies. Only 5.3% of the participants had a recent dengue infection confirmed by RT-PCR. Although the resurgence of dengue in La Réunion is very recent (2016), the rate of anti-dengue IgG positivity, a marker of past infections, was already high at 43% in this study. Dengue transmission was focal in time and space, as most cases were detected within a 100-m radius of the ICs, and within a time interval of less than 7 days between infections detected in a same cluster. No particular demographic or socio-cultural characteristics were associated with dengue infections. On the other hand, environmental risk factors such as type of housing or presence of rubbish in the streets were associated with dengue infections.

## 1. Introduction

Dengue is the most common arboviral disease in the world [1]. Half the world’s population lives in areas at risk of dengue virus (DENV) infection [2]. The burden is increasing due to climate change and increases in urbanization, population, and air traffic [3,4,5]. Globally, there are an estimated 100 million new infections per year, including 500,000 hospitalizations for severe cases and 10,000 to 15,000 deaths (2021 estimate) [1,6,7]. The epidemiological situation of dengue in Africa and the Indian Ocean region is poorly understood [8]. Seroprevalence studies of febrile travelers returning to Europe have estimated a lower prevalence of dengue in travelers from Africa than from Asia or the Americas [9,10]. However, it has been estimated that 15.7 (10.5–22.5) million symptomatic dengue infections occurred in Africa (including Madagascar and La Réunion), making Africa the second most affected continent after Asia [6].

La Réunion is a French overseas department of 2503 km^2^ with nearly 860,000 inhabitants [11]. It is located in the southwest Indian Ocean, near the east coast of Madagascar. A tropical environment and a high-income industrial development characterize this island. Dengue is transmitted by the bite of a mosquito and has no animal reservoir in La Réunion. *Aedes albopictus* is the main mosquito vector of dengue found in La Reunion [12]. Four serotypes for dengue (DENV1–DENV4) have been identified worldwide. The infection with one serotype confers lifelong immunity for it and only some cross-immunity for the other during the first months after infection. The island faced a major chikungunya epidemic in 2005–2006 with an attack rate of 38% [5]. In 1977–1978, a massive DENV2 epidemic occurred on the island with an attack rate of 30% [13,14,15]. Thereafter, only sporadic dengue cases or small outbreaks of approximatively 200 cases had been detected on the island. In 2017, the surveillance system noted an unusual persistence of dengue cases during the austral winter (June to September) likely due to warmer temperatures [16] and then an intensification of the circulation of the virus that has continued until the 2021. Most dengue cases have been detected in April and May [17]. Three serotypes have been detected, DENV1, DENV2, DENV3. The most affected sectors were the south and west of the island [17]. Table 1 provides the number of estimated and confirmed dengue cases and deaths from 2018 to 2021 in La Réunion. The mortality rate reached 0.11% in 2021 [17].

In La Réunion, following the chikungunya epidemic in 2006, a surveillance system was created in order to rapidly identify the first cases of arbovirus infections [18]. Santé Publique France is directly informed of any laboratory-confirmed case of arbovirus infections. Vector control measures are provided by the Agence Régionale de Santé (ARS) to limit the spread of the virus by spraying with insecticide and eliminating breeding sites within a defined perimeter around the residence of the identified case.

Despite very suitable access to care, a sustained surveillance system as well as dengue prevention and control policies that have been in place for more than 10 years, the dengue epidemic in La Réunion continues to be rampant. One hypothesis that could explain the persistence of the epidemic is that part of the transmission is due to asymptomatic forms of the disease thus not detected by the surveillance system. The estimated percentage of asymptomatic cases during DENV outbreaks varies between 50% and 90% [19,20,21,22,23]. Studies collecting data on the presence of DENV infections considering all disease patterns including asymptomatic people have been performed in Latin America and Southeast Asia [19,24,25,26] but not in the Indian Ocean region. Asymptomatic individuals could act as a reservoir of the virus. Knowing their prevalence is necessary for establishing public health policies for blood donor screenings, for conducting dengue vaccine clinical trials and has implications for vector control strategies.

This study aimed to assess the proportion of asymptomatic dengue infections by an active search around identified dengue index cases, households, and neighborhoods and to identify the main risk factors related to recent and past dengue infections.

## 2. Materials and Methods

### 2.1. Study Design

This cross-sectional observational study included a household-based survey covering the whole island of La Réunion. The strategy to detect dengue infections, according to a geographical cluster recruitment study design, consisted in an active search in a group of participants selected within a fixed radius of the home or workplace of an index case (IC).

### 2.2. Clinical Definitions and Laboratory Diagnosis

Dengue-like syndrome is defined as an acute fever associated with two or more of the following signs or symptoms: nausea, vomiting, rash, headache, retro-orbital pain, myalgia, arthralgia, and bleeding [27]. No standard definitions exist for “pauci-symptomatic” and “asymptomatic”. In the present study, “asymptomatic” infections are defined as the complete absence of symptoms after a follow-up period of 14 days and the “pauci-symptomatic” include the symptomatic infections that do not meet the criteria for a dengue-like syndrome.

### 2.3. Laboratory Diagnosis

The World Health Organization (WHO) recommendations for the diagnosis of dengue include enzyme-linked immunosorbent assay (ELISA)-based detection of dengue-specific IgM antibodies or a ≥4-fold increase in the titer of total antibodies to DENV in paired acute and convalescent sera; or detection of DENV by reverse transcription polymerase chain reaction (RT-PCR) in plasma, serum, or whole blood [28]. According to the kinetics of DENV infection markers in the blood, anti-dengue IgM antibodies are detectable in the blood approximately three months after the infection, and anti-dengue IgG antibodies are lifelong markers [29]. Molecular detection of the virus through RT-PCR is possible during the first five days of the disease. The plaque reduction neutralization test (PRNT) is a serological test, more sensitive and specific than the ELISA method for dengue diagnosis. Moreover, PRNT can be used to identify the infecting serotype in primary infection. Furthermore, this technic was too expensive and labor consuming to be retained for our needs [30]. In the present study, the three markers (IgM, IgG (ELISA), and RT-PCR) were measured in the blood to detect recent or past dengue infections among people living in the neighborhood of dengue cases.

### 2.4. Identification of Index Cases

The ICs were recruited among patients who consulted the emergency rooms of the North or South University Hospital or their general practitioners with dengue-like symptoms and in whom dengue was confirmed by biological examination in hospital or city laboratories. The selection criteria for ICs were individuals ≥ 12 months old, with a positive dengue RT-PCR or a positive IgM, and with a dengue-like syndrome. An IC could not be located within 400 m of an already included IC to avoid overlap. IC was notified to the principal investigator of the study in the 14 days following their lab-confirmed infection and then included in the study by the field team as soon as possible according to the team capacity. The rationale for this time interval was to try to be close to the infection day of the IC while being constrained by logistical considerations of field recruitment. The date of inclusion of the IC was the date of commencement of screening and inclusion of study participants in the IC household and neighborhood forming a cluster. The duration for including participants in a cluster was of two weeks after the date of inclusion of the IC.

### 2.5. Sample Size

The sample size was targeted according to the capacity of the study team and laboratory and estimated a population density of approximately 100 people within a 100-m radius of the household of the IC. Estimating that 40% of individuals would be absent at the time of the survey and that 20% of individuals would refuse, a target of 40 participants enrolled per cluster seemed realistic. Considering an estimated prevalence of dengue in La Reunion of 3% and that in similar studies using a geographical cluster design around ICs, between 4% and 27% of the participants screened had asymptomatic dengue infection [26,31]. A sample size of 600 participants would likely provide enough power (with a confidence level of 1.96 (95%) and an error of 5%), i.e., a minimum of 15 ICs were required.

### 2.6. Cluster Definitions and Data Collection

The residence of an IC represented the center of a geographic cluster. The participants were recruited from households that were within 100 m of the house of an index case. The rationale behind this 100 m was that *Ae. albopictus* mosquitoes have been shown to have limited flight ranges in Reunion [32]. This radius could be increased to 200 m if the targeted sample size was not achieved due to absences or refusal. All houses in the cluster were identified with *Google Earth* and *OpenStreetMaps* and visited for inclusion in the study. Data collection started with the household of the IC and then continued in the neighboring houses. In each household, all consenting individuals were eligible to participate, with the intent to include approximately 40 participants in each cluster. The exclusion criteria were age under one year old and any contraindication to proceeding to blood sampling.

A complete medical history including a collection of signs and symptoms by organic system was conducted.

Blood samples were collected in dry and with anticoagulant (EDTA) tubes: 1 dry tube of 4 mL for anti-DENV IgM/IgG serology (*Panbio ^TM^ Dengue IgG, IgM Capture ELISA*); 1 EDTA tube of 4 mL for DENV RT-PCR (in-house technique [33]). Urine samples were also collected and frozen for further search of the excretion of dengue virus by RT-PCR. All analyses were conducted at the laboratory of the North University Hospital. Serotypes were determined with a second RT-PCR (in-house technique of the *Centre National de Référence (CNR) Arbovirus de Marseille*). Participants reporting a dengue diagnosis in the weeks preceding the study were asked to provide their lab analysis report to confirm RT-PCR positivity for DENV. If they did not have the report, the study team requested it directly from the laboratory or from the general practitioner. Due to the lack of specificity of anti-dengue IgM for asymptomatic cases, a follow-up visit was held 15 to 21 days after the first home visit for participants with positive IgM to provide paired serology and look for IgG seroconversion or an increase in total antibody titers. At this visit, blood was only collected for anti-DENV IgM/IgG serology.

By going in the field and recruiting participants who lived in the neighborhood of identified dengue cases, the study team had the opportunity to collect, in addition to clinical data, data on socio-demographic characteristics of households as well as environmental potential risk factors present in the neighborhoods and to evaluate their possible impact on the transmission of dengue.

### 2.7. Statistical Methods

The proportions of the sampled population with DENV infections confirmed by RT-PCR and with IgG DENV-positive results were selected respectively as primary and secondary outcomes. Signs and symptoms were recorded in DENV RT-PCR-positive participants and ICs; proportions were computed and cases were classified accordingly as symptomatic (presence of fever only or with one or more other symptoms), pauci-symptomatic (absence of fever but presence of one or more other symptoms) or asymptomatic (absence of symptoms) infections. The comparison between groups of categorical variables were made using the chi-square or Fisher exact test according to the sample size. The association between explanatory variables and outcomes (positive DENV RT-PCR, positive anti-dengue IgG) was estimated using bivariate analyses and multivariate logistic regression models. The multivariate logistic regression models were constructed with the variables that showed significant association in the bivariate analysis and the best models were selected following a backward stepwise procedure of selection of variables. All analyses were performed with R Statistical Software (v.4.2.2 R Core Team 2022).

### 2.8. Ethics and Confidentiality

This study was carried out in accordance with the law n 2012-300 of 5 March 2012 relating to research involving the human person, as well as in accordance with the Good Clinical Practices [34] and the Helsinki declaration, and the participants or the parents of minors participating in the study provided written informed consent before inclusion. The study was accepted by the Comité de Protection des Personnes of the University Hospital of Saint-Etienne (CPP SUD-EST I), France (n ID RCB:2018-A02357-48).

## 3. Results

**Figure 1:**
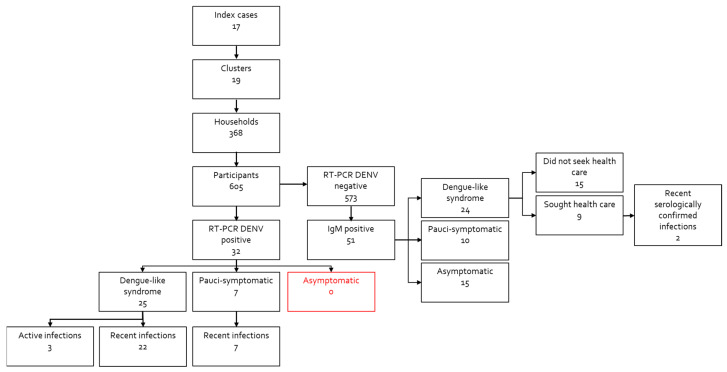
Flowchart describing the included study population and the dengue RT-PCR and anti-dengue IgM results.

The study was conducted between October 2019 and August 2020. A total of 17 dengue index cases (IC) were identified, and 605 participants were recruited from 368 households within a 100 to 200 m radius of the IC’s homes (Figure 1).

### 3.1. Cluster Investigation

**Figure 2:**
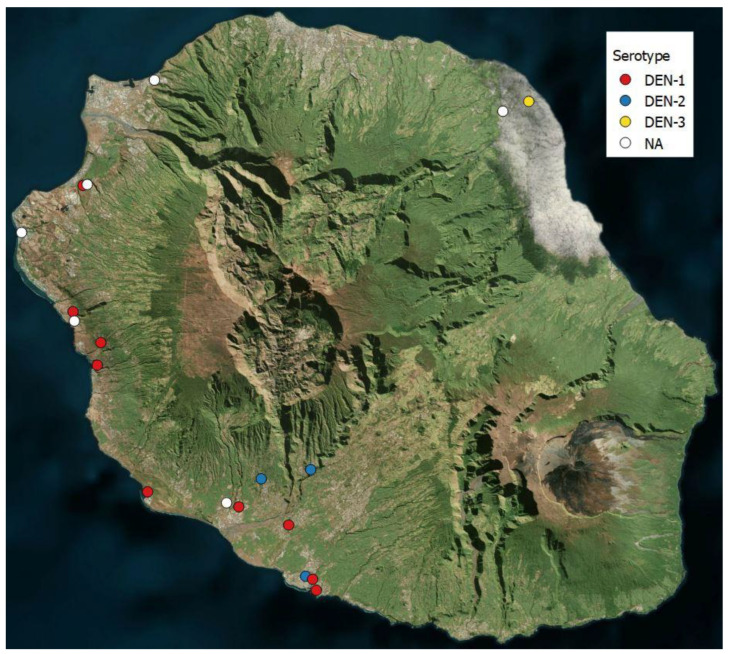
Map of the 19 clusters throughout the island. The dots represent the locations of clusters. The colors represent the serotype found in the cluster, as listed in Table 2.

Seventeen ICs formed the basis for 17 clusters. Additionally, the place of work of one IC was used to create an additional cluster, and one cluster was formed for an IC who did not return the consent form, so while the data of the IC were not used, the cluster was kept. This resulted in 19 clusters. The clusters, distributed throughout the whole island, are described in Table 2, with the prevalence of dengue infections detected by RT-PCR per cluster and the seroprevalence of IgM and IgG. The clusters are mapped in Figure 2.

### 3.2. Demographic Characteristics

Table 3 shows the demographic and health characteristics of all the study subjects. Eighty-six percent were adults, and the mean age was 46 years old. Thirty percent were more than 60 years old. Fifty-seven percent were female.

### 3.3. Detection of Dengue Virus by RT-PCR

No active asymptomatic infections confirmed by RT-PCR were detected. Out of the 605 participants recruited around the index cases, only 5.3% (32/605) presented a recent or active dengue infection confirmed by RT-PCR: only 3 were detected during the field survey, and the other 29 corresponded to confirmed recent infections reported by the participants and for which the study team could retrieve the lab results. These recent infections occurred in the 3 months preceding the survey, corresponding to the duration of IgM persistence in blood after infection. Participants with recent dengue infections were found in 10 of the 19 clusters investigated. The active dengue infections were found in only two clusters (in the south in February and in the East in March). The prevalence of recent dengue infections confirmed by molecular analysis (RT-PCR) in each cluster varied from 0% to 18% (Table 2).

### 3.4. Dengue-Positive RT-PCR in Households

The 622 study subjects (IC and participants) were distributed in 368 households. The 17 ICs and the 32 RT-PCR-positive participants were distributed across 44 households. In 17/44 households, only one study subject was included in the study, and in 27/44 households, multiple study subjects were included per house (range 2 to 5). Among these 27 households, only 2 contained more than one detected dengue infection.

### 3.5. Time Interval between Dengue Infections within Clusters

**Figure 3:**
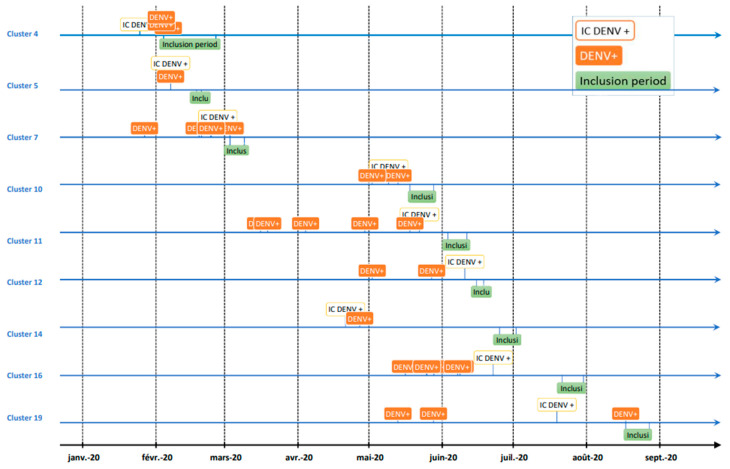
Timelines displaying the occurrence of dengue-detected infections in clusters and the inclusion period.

Figure 3 shows the timelines of dengue infection onset and the inclusion study periods for each cluster where dengue cases were detected. Dengue recent infections are clustered close in time and generally just after the onset of IC infection. The study team’s inclusion period occurred after the clustered dengue cases. Table 4 shows the time intervals between the date of laboratory confirmation of dengue infection and the date of the study team visit to include the IC and screen and include the participants in the neighborhood. For clusters 14, 16, and 19, the time interval for inclusion of the IC was not respected due to inaccurate information received at the time of screening. These data were not excluded as the deviation did not prevent from interpreting them. In clusters 4, 7, and 9, active dengue infections were detected during the field visit to recruit participants. This explains the time interval of ‘0′ days. For all the other clusters, participants with recent dengue infections, already diagnosed by general practitioners before our field visit, were included. The date of these infections could precede the infection of the ICs. These findings suggest that, for most of the clusters, the dengue virus circulated before the infection of the IC. Moreover, the infections detected in a cluster occurred in a limited period of time. Looking at the dates of these recent dengue infections, it resulted that, in the majority of the clusters, for each infection, another occurred within 7 days.

### 3.6. Dengue Serotypes

For the three active dengue infections detected within the study, serotyping was performed and resulted in one DEN-1 in cluster 4 (south) and two DEN-3 in cluster 7 (east). For ICs and participants with recent infections detected prior to the recruitment team’s visit, serotyping results were not always available. Some could be performed on residual blood samples held by the diagnosing laboratories, and for seven cases, dengue virus could be detected in a urine sample, and serotyping was performed. In the end, serotypes were available for 16/48 dengue infections. As presented in the last column of Table 2, DEN-1, DEN-2, and DEN-3 were detected, with a strong majority of DEN-1. DEN-3 was only detected in the eastern region, and DEN-2 was only found in the south. In clusters 4, 7, and 12, several serotype results were available, but only one serotype was present per cluster. Unfortunately, serotype results from the two households with multiple dengue infections were not available.

### 3.7. Clinical Presentation of Dengue RT-PCR Confirmed Infections

By gathering the 16 ICs diagnosed with RT-PCR (excluding one case detected with serology), the 27 recent dengue infections included in the study, and the 3 active dengue infections, the total number of dengue infections confirmed by RT-PCR was 48. Among these, 30 (83%) presented a dengue-like syndrome, and 8 (17%) were pauci-symptomatic, which means that symptoms were declared but not meeting the definition of a dengue-like syndrome (described in the method). Overall, the pauci-symptomatic participants did not present fever but other symptoms. Of the three cases detected by the study team, all had typical dengue presentations, and one was pre-symptomatic at the time of recruitment, reporting only intense fatigue but declared a fever the day after.

The symptoms of the 48 dengue infections are summarized in Table S1 of the supplementary material. The accuracy of these results may suffer from some memory bias as the infections of some included participants could go back 3 months. The main symptoms of dengue infections were a severe fever and severe and prolonged fatigue. The other most frequent symptoms were anorexia, headache, myalgia, and arthralgia. All these symptoms, except for anorexia, are part of the dengue-like syndrome definition. Less than 10% of dengue-confirmed study subjects complained of bleeding signs. The other pathognomonic symptoms of dengue, which are rash and retro-orbital pain, were present in 42% and 33% of study subjects, respectively. Besides fatigue, which had a mean duration of 11 days, all other symptoms had mean durations of 3 to 5 days.

### 3.8. Detection of Anti-Dengue IgM and IgG Antibodies in Blood Samples

As shown in Figure 1, anti-dengue IgM was detected in 8% (51/573) of participants with negative dengue RT-PCR results. Among these, 42 (81%) also had anti-dengue IgG antibodies. Additionally, 24 of them (47%) presented dengue-like symptoms, half of which (15/24) did not seek dengue laboratory confirmation (likely to be undiagnosed dengue infections). Ten of those with IgM (19%) were pauci-symptomatic, and 15 (29%) did not report any symptoms in the 3 months preceding the blood collection. Among the symptomatic cases, all were IgG-positive either at the first visit (37) or had seroconverted by the second visit 15 days later (2). Among the 15 cases that did not present any symptoms, 6 of them had no IgG at the first visit. Among these, three participated in a second visit: one was seroconverted, and two remained IgG negative, implying that the presence of IgM at the first visit might not be due to dengue infection. The proportion of possible asymptomatic dengue infections detected by IgM (15) accounts for 15% of all the 100 possible dengue infections (including ICs (17), recent infections (29), active infections (3), and IgM-positive participants (51)). However, this result is overestimated as it is likely that not all of the asymptomatic participants with positive IgM results were true dengue cases, as there is some lack of specificity in the dengue IgM assay performed with the ELISA technique [30]. Moreover, considering that IgM could persist for more than 3 months in some individuals, these results are difficult to interpret [35].

A total of 43% of participants had the presence of anti-dengue IgG, 42% (109/258) of which were not associated with a history of dengue, which means that 18% (109/605) of the study population had IgG with no history of dengue.

As shown in Table 1, the proportion of the population with IgG anti-dengue antibodies varies greatly between clusters, ranging between 12% and 71% of participants investigated with positive anti-dengue IgG in a cluster. The clusters with the highest prevalence of IgG-positive participants were located in the west part of the island, in the municipality of Saint-Paul, and in the south, in the municipality of Saint-Louis.

### 3.9. IgG Anti-Dengue Antibodies in Households

In 165/368 households, more than one study subject was tested (range 2 to 7). In 106/165 (64%), at least one subject had positive IgG anti-dengue antibodies. In 45/106 (42%) households, more than 50% of the study subjects living in the household were IgG-positive. In 35/106 (33%), all study subjects included were IgG-positive.

### 3.10. Risk Factors for Dengue Infection Confirmed by RT-PCR and Parameters Associated with Past Dengue Infection Detected by the Presence of Anti-Dengue IgG

Table 5 lists the explanatory variables that were first cross-analyzed with two outcomes: RT-PCR DENV-positive and IgG anti-dengue-positive. “History of dengue”, “smoking”, “earthen courtyard floor”, “presence of windows with glasses”, “presence of rubbish in the surrounding area”, and “farm in the surrounding area” showed a significant association with a positive RT-PCR DENV result. The variable “History of dengue” was not introduced in the multivariable model as the significance was due to a recruitment bias since most RT-PCR dengue-positive study subjects knew their diagnosis at the time of their study inclusion. The multivariate logistic regression model identified three explanatory variables significantly associated with the outcome of RT-PCR dengue-positive, which are listed in Table 6 with their adjusted odds ratios. Several variables were associated with the outcome of IgG anti-dengue-positive in the bivariate cross-analysis (Table 5). However, many of them were linked to the “age” of the study subjects. Indeed, when adjusting them in the multivariate logistic regression model, only three variables remained significantly associated with the outcome (Table 6). The bivariate analysis and the multivariate logistic regression models did not show any socio-demographic criteria (neither activity, nor level of education, nor comorbidity) as risk factors for dengue infections. Only environmental factors, such as the type of housing and the presence of waste in the environment, were risk factors associated with dengue infection.

## 4. Discussion

In 2019, the dengue outbreak in La Réunion had more than 18,000 confirmed cases. Among these, 17 were included as index cases for the study, and the study team surveyed a perimeter of 100–200 m around their households or working places looking for identified, undiagnosed, or asymptomatic dengue infections. The findings suggest that a very small proportion of the population presented asymptomatic forms of dengue. Indeed, confirmed dengue cases (PCR or serology) were only detected in 5.3% of the study population during the survey or 3 months prior to the survey, and all were symptomatic cases. These results should be interpreted with caution because our study design allowed us to obtain only part of the picture. We were able to collect data over a short period of time for each cluster, and the inhabitants of the cluster area were only partially represented. Therefore, we may have missed some cases. The value of IgM testing is that it provides a broader picture of dengue circulation. The fact that we found only a maximum of 15% of possible asymptomatic recent dengue infections detected by the presence of anti-dengue IgM supports the idea that asymptomatic cases do not appear to be numerous. Although the re-emergence of dengue in La Réunion is very recent (2016), the rate of IgG anti-dengue positivity, markers of past infections, has already reached 43%. In the literature, the frequency of DENV infections detected around an IC using a geographic cluster design ranges from 4% to 27% [26,31]. With the 5.3% prevalence found in this study, La Réunion is located in the lower part of this range. The prevalence of anti-dengue IgG was high, and 42% of the IgG-positive participants could not recall a past dengue infection. Moreover, the multivariate analysis identified an association between a past infection of chikungunya and the presence of anti-dengue IgG. Some past dengue infections could have been classified as “chikungunya” cases as the latter was well known by the population after the important outbreak of 2005. On the one hand, 30% of participants with no history of dengue infection had IgG-positive results, and on the other hand, 24% of participants with a history of dengue infection did not have any IgG, which is a lifelong marker of past disease. These discordant results reflect the likely memory bias and that dengue clinical presentation is not specific and challenging to confirm without testing.

Because of the four serotypes of dengue, no herd immunity could be expected, mainly as only dengue 1 and dengue 2 have circulated in the whole island, whereas dengue 3 only circulated on the eastern coast, and no dengue 4 serotype was identified yet [17].

We detected only 15 (15%) undiagnosed probable dengue infections, defined as participants who presented a dengue-like syndrome in the 3 months preceding our visit and the presence of IgM but who did not seek health care. La Réunion is a high-income island with very suitable access to health. Universal health care is largely financed by government national health insurance, and people, therefore, easily seek care to obtain medicine or time off work. In fact, we found many confirmed dengue infections that occurred before our visit.

Within cities such as Saint-Pierre, for example, the proportion of IgG across clusters differed widely. This illustrates that the dengue virus seems to circulate most often in the same places and that the geographical range of transmission could be very limited (less than 200 m of radius).

To the best of our knowledge, the usual transmission rate in a household is not known. In our study, 20% of the households visited contained only one inhabitant, and in 44% of the households that contained more than one inhabitant, only one member agreed to be included in the study. Although we were not able to include all members of every household visited, we have data on 27 households containing dengue infections in which more than one participant was included. Only 2 households contained more than one infection, and in 25 households with multiple inclusions, only one household member had a recent dengue infection. These data suggest that the rate of transmission in households is probably not that high.

In the present study, we follow a geographic cluster recruitment design. We needed an initial case to allow us to identify in which neighborhoods it would be worthwhile to include asymptomatic participants to look for infection. This first case was called an index case because it represented an approximation of dengue circulation in a neighborhood, but in reality, it did not mean that it was the index case of an epidemic. This is well illustrated in Figure 3, where the timeline highlighted that the IC for the study was not the first case in a neighborhood transmission outbreak. Interestingly, this timeline shows that the inclusion period of the study team occurred mostly after the occurrence of a few cases of clustered dengue. Moreover, the discovery of active infections during the inclusion period was rare; only three active infections were detected during the entire study period. This underscores the need for very reactive action to detect circulating cases.

Although none of the demographic nor socio-economic variables studied in our participants were found to be associated with dengue infection, environmental risk factors such as the type of housing or the presence of rubbish in the streets were associated with dengue infections. Considering that dengue is a vector-borne disease, thus transmitted by the bite of a mosquito, it seems reasonable that the environment has an impact on transmission, as observed in our results. As shown in Table 6, the presence of a courtyard, farm, or rubbish in the neighborhood is a predictor of dengue infections. This may be simply because these locations provide ample breeding opportunities for mosquitoes.

Our main result, the proportion of dengue infection found in the clusters, is consistent with previous studies. However, unlike most observational epidemiological studies on dengue, we did not find a large proportion of asymptomatic cases. We can put forward several hypotheses to try to explain this important difference between the proportion of asymptomatic cases generally described in the literature and our results. First, our recruitment method with a single inclusion visit with blood sampling (and a second visit only for IgM-positive cases) provided us only a limited view of the potential viremia of our participants; a design with repeated sampling to look for the presence of the virus would have been more likely to detect cases (but hardly acceptable to the participants). Furthermore, the serology provided a larger vision in time and could have signaled the presence of asymptomatic infections that occurred before our venue. Only 15% of possible recent asymptomatic dengue infections detected thanks to positive anti-dengue IgM were found, which is still far below the expected 50% to 90%. Second, we included only 7% of children, and according to the literature, the main victims of dengue in endemic areas are children. The data to establish a 50–90% asymptomatic rate come mainly from studies in children [36,37,38,39,40,41,42]. We can hypothesize here that asymptomatic forms are rarer in non-immune adults. Moreover, on Reunion Island, the re-emergence of dengue is relatively recent (2016), and only two serotypes have been widely circulating on the island. The level of immunity in the population, 43% IgG-positive, should be essentially monotypic. The role of immunity in the clinical presentation of the disease is widely discussed in the literature with no absolute consensus except for secondary infections that may be likely more severe [19]. Finally, the high rate of asymptomatic infections in the literature may rather correspond to undiagnosed infections as in endemic countries, people accustomed to the occurrence of dengue do not necessarily seek care. Moreover, most observational studies on dengue transmission were conducted in low- and middle-income countries where dengue was highly endemic. Our results are interesting as it is one of the first studies on dengue transmission in a high-income country where dengue is emergent. The external validity of our results should be evaluated in countries with similar characteristics of high income, suitable access to health care, and the emergence of the disease.

The implications of these results for public health are that, firstly, as the proportion of asymptomatic dengue infections and undiagnosed infections in La Réunion appears to be low, the data provided by the health authorities are likely a suitable estimation of reality. Second, the same neighborhoods seemed more affected both by a high prevalence of anti-dengue IgG and by active infections, suggesting that known areas of dengue transmission could benefit from targeted prevention actions ahead of the outbreak. Third, the focal nature of dengue in place and time, illustrated by the short time interval between detected infections within clusters, as already described in previous studies, reinforces the need for very rapid and localized actions. Studied clusters in the same city showed different levels of IgG prevalence; therefore, prevention actions should be considered by neighborhood and not by city. Fourth, the analysis failed to demonstrate any association between dengue infection risk and education level or other demographic characteristics. Only environmental variables were significant, which shows the importance of cleaning work in the neighborhoods in addition to prevention messages. Moreover, the knowledge of the population about dengue is very suitable, as demonstrated in a previous qualitative study [43].

The design of recruitment had several limitations in detecting asymptomatic dengue infections. The proportion of houses within 200 m of the IC from which participants were included in the study was low, less than 50% across all the clusters. Many houses visited were empty during usual working hours. Indeed, the proportion of women and retired people is high in our study population. This reflects a high proportion of non-working women in La Réunion [11]. However, the proportion of workers on the island was 55% in 2019, according to INSEE data [44]. In the present study, the proportion of workers among the adult population reached 39% despite the constraint of recruitment during weekday working hours. In the houses where people were present at the time of our visit, the acceptability was globally suitable, and the local population showed great willingness to participate in the research. However, refusal was common for children as our study procedures included venipuncture. Despite the low proportion of houses included in the study, dengue cases confirmed with RT-PCR were detected in half of the clusters investigated.

According to the study protocol, the interval between the confirmation of the dengue infection and the study visit was meant to be within 15 days. However, due to challenges faced during the field recruitment work, this interval of 15 days was not respected for three index cases and ranged between 30 and 71 days. This increased delay may have reduced our capability to detect dengue infections.

Due to the COVID-19 pandemic that occurred during the recruitment period, we had to restrict the study procedures for safety reasons and were not able to proceed to all of the initially planned second visits for participants who had a positive anti-dengue IgM result. These IgM results had to be interpreted with caution as they could not be correlated to a clinical presentation nor to an IgG seroconversion.

Further research is needed as detecting active asymptomatic dengue infections is a real challenge given the likely short duration of viremia and the very focal duration of dengue outbreaks in neighborhoods. Testing would need to take place much more widely and repeatedly, which may not be acceptable to the participants, especially if they do not suffer from any symptoms. It could be interesting to consider urine testing to detect dengue infection by molecular analysis. This is a non-invasive test and, thus, more acceptable. We demonstrated the possibility of detecting the virus in urine a few days after infection [data not yet published]. Further research will be helpful to complete and refine these results to improve public health policies for dengue control in countries in the Indian Ocean region.

## 5. Conclusions

The proportions of asymptomatic and undiagnosed dengue infections are very low in La Réunion. Dengue transmission is focal in time and place, and environmental factors arising from human life are the principal risk factors. Public health prevention actions should be highly targeted.

## Figures and Tables

**Table 1:** Morbidity and mortality data due to dengue from 2018 to 2021 in La Réunion [17].

Date	Estimated Cases	Confirmed Cases	Death
2018	15,460	6770	6
2019	42,420	18,217	14
2020	30,580	16,414	22
2021	59,230	29,577	33

**Table 2:** Locations of the clusters and number of participants included with DENV RT-PCR and anti-dengue IgM- and IgG-positive results (results of IC are not included). In clusters where no DENV RT-PCR-positive participants were detected, the serotype of the IC is displayed.

Cluster	Season [Month]	Location	Number of Participants	DENV RT-PCR+	IgM +	IgG +	Serotype
				*n* (%)	*n* (%)	*n* (%)	
1	Winter [10]	South	17	0	0	2 (12%)	DEN-2
2	Summer [11]	South	33	0	5 (15%)	14 (42%)	DEN-2
3	Summer [11]	South	43	0	2 (5%)	21 (49%)	DEN-1
4	Summer [02]	South	39	3 (8%)	6 (15%)	19 (49%)	DEN-1
5	Summer [02]	South	39	1 (3%)	1 (3%)	11 (28%)	DEN-2
6	Summer [02]	South	46	0	3 (7%)	19 (41%)	DEN-1
7	Summer [03]	East	33	6 (18%)	5 (15%)	15 (45%)	DEN-3
8	Summer [03]	East	27	0	3 (11%)	7 (26%)	NA
9	Summer [04]	South	3	0	0	1 (33%)	DEN-1
10	Winter [05]	South	31	2 (6%)	3 (10%)	5 (16%)	DEN-1
11	Winter [06]	South	45	5 (11%)	14 (31%)	32 (71%)	NA
12	Winter [06]	West	16	2 (12%)	7 (44%)	8 (50%)	DEN-1
13	Winter [06]	West	13	0	0	2 (15%)	DEN-1
14	Winter [07]	West	12	2 (17%)	3 (25%)	6 (50%)	NA
15	Winter [07]	West	49	1 (2%)	4 (8%)	28 (57%)	NA
16	Winter [07]	West	56	7 (13%)	6 (11%)	29 (52%)	DEN-1
17	Winter [08]	West	28	0	1 (4%)	6 (21%)	DEN-1
18	Winter [08]	West	12	0	2 (17%)	6 (50%)	NA
19	Winter [08]	West	63	3 (5%)	1 (2%)	27 (43%)	NA
Total			605	32 (5.3%)	66 (11%)	258 (43%)	

**Table 3:** Demographic characteristics.

Demographic Characteristics	Index Cases N = 17	Participants N = 605
Sex		
Female	11 (65%)	346 (57%)
Male	6 (35%)	259 (43%)
Age mean (SD); median [IQR]	36 (21); 36 [25]	46 (20); 48 [30]
Age categories (years)		
<5	1 (6%)	5 (1%)
5–11	3 (18%)	36 (6%)
12–17	0	39 (6%)
18–59	11 (65%)	340 (56%)
≥60	2 (12%)	185 (31%)
Activity		
Housewife/husband or unemployed	1 (6%)	135 (22%)
Retired/Disabled	1 (6%)	160 (26%)
Student/in training	4 (24%)	93 (15%)
Worker	11 (65%)	205 (34%)
NA	0	12 (2%)

SD: standard deviation; IQR: interquartile range; NA: not available.

**Table 4:** Time interval in days between the date of laboratory confirmation of dengue infections and the date of the study team field visits to screen and include ICs and participants.

Cluster no	Time Interval between the Date of Dengue Infection Confirmation and the Date of Inclusion in the Study [days]		Minimum Time Interval between Dengue Infections Dates in Each Cluster [days]
	**IC**	**Participants**	
Cluster 01	4	-	-
Cluster 02	8	-	-
Cluster 03	7	-	-
Cluster 04	10	0, 7, 9	0–9
Cluster 05	12	13	0
Cluster 06	11	-	-
Cluster 07	13 *	0, 12, 0, 10, 13, 36	0–23
Cluster 08	14	-	-
Cluster 09	0 ^&^	-	-
Cluster 10	9	8, 25	4–7
Cluster 11	13	22, 86, 84, 43, 68	3–19
Cluster 12	5	22, 46	14–25
Cluster 13	12	-	-
Cluster 14	71	65	6
Cluster 15	NA ^µ^	36	-
Cluster 16	31	57, 54, 54, 44, 45, 73, 64	1–14
Cluster 17	13	-	-
Cluster 18	NA ^#^	-	-
Cluster 19	30	81, 105, 9	15–29

* all diagnostics were confirmed by RT-PCR except for the IC of cluster 07, which was confirmed by the presence of IgM. ^&^ the dengue confirmation by RT-PCR was made by the study team on the day of inclusion, ^µ^ the IC could not be included in the study because the signed informed consent was not returned. ^#^ there is no IC for cluster 18, the center of the cluster was the working place of IC 17.

**Table 5:** List of explanatory variables cross-analyzed with the outcomes RT-PCR DENV-positive and IgG-positive and the associated *p*-value (Wilcoxon rank sum test; Fisher’s exact test; Pearson’s chi-squared test).

Explanatory Variables	Outcome					
	RT-PCR DENV-Positive			IgG-Positive		
	OR ^#^	95%CI	*p*-Value	OR ^#^	95%CI	*p*-Value
Age categories			0.3			>0.9
Sex			>0.9			0.7
Body mass index (BMI) categories			0.3			0.3
**Duration of stay home ≥ 20 h a day**			0.5	**2.78**	1.7-4.7	**<0.001**
**Activity: student/in training**			0.2	**0.3**	0.2–0.5	**<0.001**
**History of dengue**	**28**	8.8–144	**<0.001**	**7.5**	5–11.5	**<0.001**
**History of chikungunya**			0.8	**3.7**	2.6–5.5	**<0.001**
**Level of education**						
**Never attended school**			0.5	**5.2**	1.5–23.9	**0.015**
**Primary school**			>0.9	**2**	1.2–3.4	**0.005**
**Secondary school**			0.5	**1.6**	1.1–2.5	**0.031**
**High school**			0.13	0.8		0.2
Yellow fever vaccination			0.6			0.11
**Smoking**	**0.09**	0.002–0.6	**0.001**			0.8
**Risky drinking ^$^**			0.6	**1.2**	0.4–4.2	**0.017**
**Chronic disease**			0.11	**1.4**	1–2	**0.04**
**Chronic medication**			0.7	**1.5**	1.1–2.1	**0.017**
Sense of severity			0.2			0.7
Sense of risk ^&^			0.5			0.2
Mosquito protection use ^¥^			0.8			0.8
Mosquito bites ^α^			0.4			0.11
Mosquito presence			0.2			0.074
Season			0.2			0.2
**House with courtyard**			0.2	**4.6**	2.4–9.9	**<0.001**
Type of courtyard			>0.9			0.2–0.9
**Surface of courtyard**						
**100–500 square meters**			0.5	**1.8**	1.2–2.6	**0.004**
**>500 square meters**			0.5	**2**	1.3–3	**0.003**
**Earthen courtyard floor**	**2.1**	1–4.4	**0.032**	**1.8**	1.2–2.9	**0.009**
**Presence of grass**			0.3	**1.5**	1–2.1	**0.04**
**Presence of windows with glasses**	**0.5**	0.2–1	**0.032**	**0.5**	0.3–0.9	**0.007**
**Presence of air conditioning**			0.6	**0.6**	0.5–1	**0.032**
**Presence of swimming-pool**			>0.9	**0.3**	0.1–0.8	**0.007**
Presence of stagnant water			0.7			0.6
**Presence of animals**			0.8	**1.9**	1–3.4	**0.028**
**Presence of poultry**			0.076	**2**	1.3–3.2	**0.001**
Presence of rubbish in the courtyard			>0.9			0.12
**Presence of rubbish in the surrounding area**	**1.8**	0.9–3.5	**0.043**	**1.8**	1.2–2.6	**0.002**
Orchard in the surrounding area			0.12			0.8
**Banana plantation in the surrounding area**			>0.9	**0**	0–0.4	**0.002**
Sugarcane plantation in the surrounding area			0.4			0.14
Other agriculture in the surrounding area			0.2			0.12
**Farm in the surrounding area**	**5.6**	1.5–17.9	**0.006**			0.051
Seaside			0.6			0.2
Industrial area			>0.9			0.4
**Neighborhood of houses**			0.8	**1.7**	1.2–2.6	**0.003**
**Neighborhood of buildings**			0.6	**0.5**	0.3–0.8	**0.003**

^#^ OR: odd ratios are displayed only for explanatory variables that presented a *p*-value < 0.05. ^$^ A risky drinking is defined as more than two drinks per day. ^&^ The question behind the “sense of risk” variable was the estimated probability (low, high, very high) of contracting the disease in the next five years. **^α^** Feeling like you are frequently bitten by mosquitoes. ^¥^ Mosquito-repellent body lotion

**Table 6:** Significant (*p* < 0.05) adjusted ORs resulting from the multivariate logistic regression models associating the significant explanatory variables from the bivariate cross-analysis to the outcomes RT-PCR DENV-positive and IgG anti-dengue-positive.

Explanatory Variables	aOR	95%CI	*p*-Value
	Outcome: RT-PCR DENV-positive		
Smoking	**0.08**	**0.0–0.4**	**0.02**
Earthen courtyard floor	**2.1**	**1–4.3**	**0.046**
Farm in the surrounding area	**5.5**	**1.6–16.9**	**0.005**
Presence of rubbish in the surrounding area	1.6	0.3–1.12	0.20
Presence of windows with glasses	0.5	0.8–3.1	0.09
	Outcome: IgG anti-dengue-positive		
History of chikungunya	**2.5**	**1.6–3.9**	**<0.001**
Type of housing: house with courtyard	**5.3**	**2.2–14**	**<0.001**
Presence of rubbish in the surrounding area	**1.6**	**1.1–2.6**	**0.04**
Duration of stay home ≥ 20 h a day	1.7	0.9–3.3	0.15
Activity: student/in training	0.4	0.1–1.7	0.30
Level of education			0.07
Never attended school	2.2	0.5–15.9	
Primary school	1.6	0.7–3.6	
Secondary school	0.9	0.6–1.8	
High school	0.6	0.3–1.1	
Surface of courtyard			0.26
100–500 square meters	1.02	0.6–1.8	
>500 square meters	1.5	0.8–2.9	
Earthen courtyard floor	1.4	0.8–2.5	0.21
Presence of windows with glasses	1.04	0.6–1.9	0.89
Presence of swimming-pool	0.7	0.3–1.7	0.44
Presence of poultry	0.9	0.5–1.6	0.81
Neighborhood of houses	0.8	0.4–1.3	0.36

## Data Availability

Data are available in the YARETA portal of the University of Geneva, https://yareta.unige.ch/home (accessed on 5 February 2023), organizational unit: DEMARE.

## Supplementary Materials

The following supporting information can be downloaded at: https://www.mdpi.com/article/10.3390/v15030742/s1, Table S1: Description and frequency of symptoms declared by the 48 dengue RT-PCR confirmed subjects.

## References

[1] Dengue and Severe Dengue. https://www.who.int/news-room/fact-sheets/detail/dengue-and-severe-dengue.

[2] Brady O.J., Gething P.W., Bhatt S., Messina J.P., Brownstein J.S., Hoen A.G., Moyes C.L., Farlow A.W., Scott T.W., Hay S.I. (2012). Refining the Global Spatial Limits of Dengue Virus Transmission by Evidence-Based Consensus. PLoS Negl. Trop. Dis..

[3] Dash A.P., Bhatia R., Sunyoto T., Mourya D.T. (2013). Emerging and Re-Emerging Arboviral Diseases in Southeast Asia. J. Vector Borne Dis..

[4] Gubler D.J. (2002). Epidemic Dengue/Dengue Hemorrhagic Fever as a Public Health, Social and Economic Problem in the 21st Century. Trends Microbiol..

[5] Flahault A. (2007). Emerging infectious diseases: The example of the Indian Ocean chikungunya outbreak (2005-2006). Bull. Acad. Natl. Méd..

[6] Bhatt S., Gething P.W., Brady O.J., Messina J.P., Farlow A.W., Moyes C.L., Drake J.M., Brownstein J.S., Hoen A.G., Sankoh O. (2013). The Global Distribution and Burden of Dengue. Nature.

[7] Stanaway J.D., Shepard D.S., Undurraga E.A., Halasa Y.A., Coffeng L.E., Brady O.J., Hay S.I., Bedi N., Bensenor I.M., Castañeda-Orjuela C.A. (2016). The Global Burden of Dengue: An Analysis from the Global Burden of Disease Study 2013. Lancet Infect. Dis..

[8] Jaenisch T., Junghanss T., Wills B., Brady O.J., Eckerle I., Farlow A., Hay S.I., McCall P.J., Messina J.P., Ofula V. (2014). Dengue Expansion in Africa—Not Recognized or Not Happening?. Emerg. Infect. Dis..

[9] Neumayr A., Muñoz J., Schunk M., Bottieau E., Cramer J., Calleri G., López-Vélez R., Angheben A., Zoller T., Visser L. (2017). Sentinel Surveillance of Imported Dengue via Travellers to Europe 2012 to 2014: TropNet Data from the DengueTools Research Initiative. Eurosurveillance.

[10] Amarasinghe A., Kuritsky J.N., Letson G.W., Margolis H.S. (2011). Dengue Virus Infection in Africa. Emerg. Infect. Dis..

[11] La Réunion—La France et Ses Territoires | Insee. https://www.insee.fr/fr/statistiques/5039941?sommaire=5040030.

[12] Boyer S., Foray C., Dehecq J.-S. (2014). Spatial and Temporal Heterogeneities of Aedes Albopictus Density in La Reunion Island: Rise and Weakness of Entomological Indices. PLoS ONE.

[13] Kles V., Michault A., Rodhain F., Mevel F., Chastel C. (1994). A serological survey regarding Flaviviridae infections on the island of Réunion (1971–1989). Bull. Soc. Pathol. Exot. 1990.

[14] Michault A. (1998). Insularity and epidemic risks in Réunion. Bull. Soc. Pathol. Exot. 1990.

[15] Zeller H.G. (1998). Dengue, arbovirus and migrations in the Indian Ocean. Bull. Soc. Pathol. Exot. 1990.

[16] DiSera L., Sjödin H., Rocklöv J., Tozan Y., Súdre B., Zeller H., Muñoz Á.G. (2020). The Mosquito, the Virus, the Climate: An Unforeseen Réunion in 2018. GeoHealth.

[17] SPF Surveillance de la dengue à La Réunion. Point au 7 décembre 2021. https://www.santepubliquefrance.fr/regions/ocean-indien/documents/bulletin-regional/2021/surveillance-de-la-dengue-a-la-reunion.-point-au-7-decembre-2021.

[18] Renault P., Solet J.-L., Sissoko D., Balleydier E., Larrieu S., Filleul L., Lassalle C., Thiria J., Rachou E., de Valk H. (2007). A Major Epidemic of Chikungunya Virus Infection on Reunion Island, France, 2005–2006. Am. J. Trop. Med. Hyg..

[19] Grange L., Simon-Loriere E., Sakuntabhai A., Gresh L., Paul R., Harris E. (2014). Epidemiological Risk Factors Associated with High Global Frequency of Inapparent Dengue Virus Infections. Front. Immunol..

[20] Wang T., Wang M., Shu B., Chen X., Luo L., Wang J., Cen Y., Anderson B.D., Merrill M.M., Merrill H.R. (2015). Evaluation of Inapparent Dengue Infections during an Outbreak in Southern China. PLoS Negl. Trop. Dis..

[21] Chikaki E., Ishikawa H. (2009). A Dengue Transmission Model in Thailand Considering Sequential Infections with All Four Serotypes. J. Infect. Dev. Ctries..

[22] Dengue—Chapter 4—2020 Yellow Book | Travelers’ Health | CDC. https://wwwnc.cdc.gov/travel/yellowbook/2020/travel-related-infectious-diseases/dengue.

[23] WHO | Dengue. http://www.who.int/denguecontrol/en/.

[24] Dussart P., Baril L., Petit L., Beniguel L., Quang L.C., Ly S., Azevedo R., do S.S., Meynard J.-B., Vong S. (2012). Clinical and Virological Study of Dengue Cases and the Members of Their Households: The Multinational DENFRAME Project. PLoS Negl. Trop. Dis..

[25] Ly S., Fortas C., Duong V., Benmarhnia T., Sakuntabhai A., Paul R., Huy R., Sorn S., Nguon K., Chan S. (2019). Asymptomatic Dengue Virus Infections, Cambodia, 2012–2013. Emerg. Infect. Dis..

[26] Duong V., Lambrechts L., Paul R.E., Ly S., Lay R.S., Long K.C., Huy R., Tarantola A., Scott T.W., Sakuntabhai A. (2015). Asymptomatic Humans Transmit Dengue Virus to Mosquitoes. Proc. Natl. Acad. Sci. USA.

[27] PAHO (2017). Tool for the Diagnosis and Care of Patients with Suspected Arboviral Diseases.

[28] WHO | Dengue Guidelines for Diagnosis, Treatment, Prevention and Control: New Edition. http://www.who.int/rpc/guidelines/9789241547871/en/.

[29] Guzman M.G., Gubler D.J., Izquierdo A., Martinez E., Halstead S.B. (2016). Dengue Infection. Nat. Rev. Dis. Primer.

[30] Chatchen S., Sabchareon A., Sirivichayakul C. (2017). Serodiagnosis of Asymptomatic Dengue Infection. Asian Pac. J. Trop. Med..

[31] García G., Sierra B., Pérez A.B., Aguirre E., Rosado I., Gonzalez N., Izquierdo A., Pupo M., Danay Díaz D.R., Sánchez L. (2010). Asymptomatic Dengue Infection in a Cuban Population Confirms the Protective Role of the RR Variant of the FcγRIIa Polymorphism. Am. J. Trop. Med. Hyg..

[32] Lacroix R., Delatte H., Hue T., Reiter P. (2009). Dispersal and Survival of Male and Female Aedes Albopictus (Diptera: Culicidae) on Réunion Island. J. Med. Entomol..

[33] Giry C., Roquebert B., Li-Pat-Yuen G., Gasque P., Jaffar-Bandjee M.-C. (2017). Simultaneous Detection of Chikungunya Virus, Dengue Virus and Human Pathogenic Leptospira Genomes Using a Multiplex TaqMan^®^ Assay. BMC Microbiol..

[34] Légifrance—Publications Officielles—Journal Officiel—JORF N° 0277 Du 30/11/2006. https://www.legifrance.gouv.fr/download/pdf?id=REqL9dEa6zHzO_9B-g8NfXy_5j9RBhfoFzUoFVjb4G4=.

[35] Chien Y.-W., Liu Z.-H., Tseng F.-C., Ho T.-C., Guo H.-R., Ko N.-Y., Ko W.-C., Perng G.C. (2018). Prolonged Persistence of IgM against Dengue Virus Detected by Commonly Used Commercial Assays. BMC Infect. Dis..

[36] Burke D.S., Nisalak A., Johnson D.E., Scott R.M. (1988). A Prospective Study of Dengue Infections in Bangkok. Am. J. Trop. Med. Hyg..

[37] Pengsaa K., Limkittikul K., Yoksan S., Wisetsing P., Sabchareon A. (2011). Dengue Antibody in Thai Children From Maternally Transferred Antibody to Acquired Infection. Pediatr. Infect. Dis. J..

[38] Chau T.N.B., Hieu N.T., Anders K.L., Wolbers M., Le Bich L., Lu Thi Minh H., Hien T.T., Hung N.T., Farrar J., Whitehead S. (2009). Dengue Virus Infections and Maternal Antibody Decay in a Prospective Birth Cohort Study of Vietnamese Infants. J. Infect. Dis..

[39] da Cunha R.V., Dias M., Nogueira R.M., Chagas N., Miagostovich M.P., Schatzmayr H.G. (1995). Secondary Dengue Infection in Schoolchildren in a Dengue Endemic Area in the State of Rio de Janeiro, Brazil. Rev. Inst. Med. Trop. Sao Paulo.

[40] Endy T.P., Yoon I.-K., Mammen M.P., Rothman A.L. (2010). Prospective Cohort Studies of Dengue Viral Transmission and Severity of Disease. Dengue Virus.

[41] Balmaseda A., Standish K., Mercado J.C., Matute J.C., Tellez Y., Saborío S., Hammond S.N., Nuñez A., Avilés W., Henn M.R. (2010). Trends in Patterns of Dengue Transmission over Four Years of a Pediatric Cohort Study in Nicaragua. J. Infect. Dis..

[42] Capeding R.Z., Brion J.D., Caponpon M.M., Gibbons R.V., Jarman R.G., Yoon I.-K., Libraty D.H. (2010). The Incidence, Characteristics, and Presentation of Dengue Virus Infections during Infancy. Am. J. Trop. Med. Hyg..

[43] Lamaurt F., De Santis O., Ramis J., Schultz C., Rivadeneyra A., Waelli M., Flahault A. (2022). Knowledge, Attitudes, Beliefs, and Practices Regarding Dengue in La Réunion Island, France. Int. J. Environ. Res. Public Health.

[44] Dossier Complet—Département de La Réunion (974) | Insee. https://www.insee.fr/fr/statistiques/2011101?geo=DEP-974.

