# Supplementary material for: Investigation of Dengue Infection in Asymptomatic Individuals during a Recent Outbreak in La Réunion
Source: Viruses. 2023 Mar 14;15(3):742. doi: 10.3390/v15030742 (PMC10058293; doi:10.3390/v15030742)
Supplement: Supplementary file 1 [file viruses-15-00742-s001.zip › viruses-2125161-supplementary.pdf]

Table S1. Description and frequency of symptoms declared by the 48 dengue RT-PCR confirmed study subjects

			n	Dengue RT-PCR positive study subjects, N = 48
**FATIGUE**	Frequency			**47/48 (98%)**
			47	
	Grade	mild		3/47 (6%)
		moderate		9/47 (19%)
		severe		35/47 (74%)
	Duration [days]*		24	**11 (8); 10 [9]**
**FEVER**	Frequency			**46/48 (95%)**
			46	
	Grade	mild		1/46 (2%)
		moderate		14/46 (20%)
		severe		31/46 (67%)
	Duration [days]*		38	5.4 (3.2); 5.0 [3.8]
**ANOREXIA**	Frequency			**39/48 (81%)**
**HEADACHE**	Frequency			**35/48 (73%)**
			35	
	Grade	mild		9/35 (26%)
		moderate		6/35 (17%)
		severe		20/35 (57%)
	Duration [days]*		21	4.3 (4.0); 3.0 [2.0]
**MYALGIA**	Frequency			**35/48 (73%)**
			35	
	Grade	mild		1/35 (3%)
		moderate		11/35 (31%)
		severe		23/35 (66%)
	Duration [days]*		23	5.57 (3.10); 5.00 [4.00]
**ARTHRALGIA**	Frequency			**28/48 (58%)**
	Duration		20	6.2 (3.0); 6.0 [4.2]
	Location	Diffuse		25/28 (89%)
			25	
	Grade	mild		2/25 (8%)
		moderate		6/25 (24%)
		severe		17/25 (68%)
**DIZZINESS**	Frequency			**28/48 (58%)**
			28	
	Grade	mild		11/28 (39%)
		moderate		9/28 (32%)
		severe		8/28 (28%)
	Duration [days]*		20	3.30 (2.66); 2.50 [3.25]
**PRURITIS**	Frequency			**27/48 (56%)**
	Grade		27	
		mild		6/27 (22%)
		moderate		7/27 (26%)
		severe		14/27 (52%)
	Duration [days]*		17	4.1 (3.1); 3.0 [6.0]
**DIARRHEA**	Frequency			**24/48 (50%)**
			24	
	Grade	mild		6/24 (25%)
		moderate		10/24 (42%)
		severe		8/24 (33%)
	Duration [days]*		19	3.0 (3.8); 2.0 [3.0]
**RASH**	Frequency			**22/48 (46%)**
			22	
	Grade	mild		2/22 (9%)
		moderate		11/22 (50%)
		severe		9/22 (41%)
	Duration [days]*		11	5.6 (5.8); 5.0 [5.0]
**VOMITTING**	Frequency			**19/48 (40%)**
			19	
	Grade	mild		10/19 (53%)
		moderate		3/19 (16%)
		severe		6/19 (32%)
	Duration [days]*		16	2.50 (2.71); 1.50 [4.25]
**ABDOMINAL PAIN**	Frequency			**17/48 (35%)**
			17	
	Grade	mild		3/17 (18%)
		moderate		9/17 (53%)
		severe		5/17 (29%)
	Duration [days]*		14	3.6 (3.6); 3.0 [2.5]

**RETRO-ORBITAL PAIN**	Frequency			**17/48 (35%)**
			17	
	Grade	mild		2/17 (12%)
		moderate		5/17 (29%)
		severe		10/17 (59%)
	Duration [days]*		10	6 (5); 4 [2]
**SORE THROAT**	Frequency			**9/48 (19%)**
			9	
	Grade	mild		2/9 (22%)
		moderate		4/9 (44%)
		severe		3/9 (33%)
	Duration [days]*		5	4.00 (1.22); 4.00 [1.00]
**DYSPNEA**	Frequency			7/48 (15%)
**HEART FLUTTER**	Frequency			6/48 (13%)
**COUGH**	Frequency			5/48 (10%)
**CHEST PAIN**	Frequency			4/48 (8%)
**BLEEDING GUMS**	Frequency			4/48 (8%)
**EPISTAXIS**	Frequency			4/48 (8%)
**RHINORHEA**	Frequency			4/48 (8%)
**ALTERED TASTE**	Frequency			3/48 (6%)
**CONJONCTIVITE**	Frequency			3/48 (6%)
**MELENA**	Frequency			2/48 (4%)
**HEMATEMESIS**	Frequency			1/48 (2%)
**HEMATURIA**	Frequency			1/48 (2%)
**BLOOD IN STOOL**	Frequency			1/48 (2%)

* Mean (SD); Median [IQR]
